# Peptide YY 3–36 attenuates trinitrobenzene sulfonic acid-induced colitis in mice by modulating Th1/Th2 differentiation

**DOI:** 10.1080/21655979.2022.2064147

**Published:** 2022-04-20

**Authors:** Zhiqiang Li, Xiaoyuan Kuang, Tao Chen, Tao Shen, Jiahong Wu

**Affiliations:** a Department of Immunology, College of Basic Medicine, Guizhou Medical University, Guiyang, Guizhou, China; b Provincial Key Laboratory of Modern Pathogen Biology, College of Basic Medicine, Guizhou Medical University, Department of Medical Parasitology, College of Basic Medicine, Guizhou Medical University, Guiyang, Guizhou, China; c Graduate School, Zunyi Medical University, Zunyi, Guizhou, China

**Keywords:** PYY 3–36, trinitrobenzene sulfonic acid, inflammation, colitis, Th1/Th2 cells

## Abstract

Peptide YY (PYY) 3–36, the main circulatory form of PYY, plays important roles in gastrointestinal motility, secretion, and absorption. However, it is unknown whether PYY 3–36 has underlying functions in colitis. The Crohn’s disease (CD)-like mouse model in which CD is induced by trinitrobenzene sulfonic acid (TNBS) was established and utilized to investigate this potential role for PYY 3–36. The results showed that the expression of colonic mucosal PYY and PYY receptors Y1, Y2, Y4 were significantly increased in mice with TNBS-induced colitis. In vitro, PYY 3–36 remarkably inhibited the production of proinflammatory cytokines tumor necrosis factor-α (TNF-α) and interleukin-6 (IL-6) from lipopolysaccharide (LPS)-induced macrophages. In vivo, a high concentration of PYY 3–36 robustly decreased the weight loss and death rate and attenuated the pathological colon tissue damage observed in mice with TNBS-induced colitis. Further studies uncovered that PYY 3–36 treatment reduced the levels of colon myeloperoxidase (MPO) and both colonic and systemic TNF-α and IL-6 observed in murine colitis. Furthermore, flow cytometric analysis showed PYY 3–36 altered the proportion of Th1/Th2 splenocytes in the disease model of colitis. Collectively, these results suggest that PYY 3–36 may be a promising candidate for the improvement of colitis, reflected by the attenuation of colon inflammatory responses observed in experimental murine colitis.

## Graphical Abstract

**Figure:**
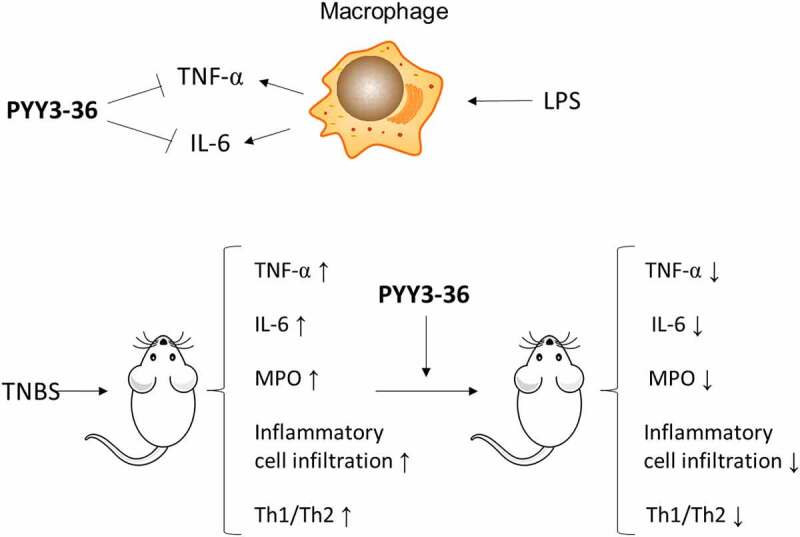

## Introduction

Crohn’s disease (CD), a common gastrointestinal disorder worldwide, is a chronic inflammatory disease of the gastrointestinal tract with symptoms, such as abdominal pain diarrhea, fever, and rectal bleeding. CD, ulcerative colitis, and microscopic colitis constitute the three main disorders of inflammatory bowel disease (IBD) [1]. Patients with CD usually experience chronic active disease or frequent relapses. The inflammation in CD caused by bowel damage and disability can occur in all segments of gastrointestinal tract [2]. The onset of CD was found to be a small peak in the second to fourth decade of human life [3]. In addition to the morbidity of this disease and no ideal curative therapies, it negatively influences the quality of life in patients with CD [1].

Currently, the etiology of CD remains unknown, but it is thought to be related to the interaction between genetic susceptibility, environmental factors, microbiota, and the intestinal immune system [2]. In particular, immunological factors were believed to exert important roles in the pathogenesis and sustainable development of CD. Some substances in food, such as emulsifiers, cause disruption of intestinal barrier function; this might contribute to bacterial dislocation [4]. Lipopolysaccharide (LPS) derived from invasive bacteria and damage-associated molecular patterns released from damaged epithelial cells initiate the intestinal immune response accompanied by changes in innate immune cells, such as macrophages. Under inflammatory conditions, monocytes from the blood differentiate into macrophages, which are recruited to the intestinal lamina propria to secrete a large amount of the proinflammatory cytokines TNF-α and IL-6 [5,6]. TNF-α-producing macrophages alter the structure and function of epithelial tight junctions and disrupt the intestinal barrier function [7]. Whereas macrophages mediate innate immune responses, T cells mediate adaptive immune responses to the microbiota in lamina propria of the gastrointestinal tract. The pathogenesis of IBD was widely hypothesized to be due to an imbalance between the subsets of CD4 + T helper (Th) cells (Th1 vs. Th2) that causes the disproportionate release of proinflammatory vs. anti-inflammatory cytokines [8]. Therefore, an increasing number of studies analyzed Th1/Th2 cytokine secretion profiles to investigate the pathogenesis of IBD. Treatments for IBD, such as mycophenolate mofetil, were evaluated for their effects on the balance between Th1 and Th2 cells [9].

Peptide YY (PYY) is a gut hormone that belongs to neuropeptide YY family. In humans, it is found in endocrine cells between epithelial cells lining the ileum, colon, and rectum [10–12]. PYY consists of 36 amino acids and has two circulating forms, PYY 1–36 and PYY 3–36. The enzyme dipeptidyl peptidase IV can cleave the amino acids tyrosine and proline of PYY (1–36) from the N-terminus of the polypeptide to produce PYY 3–36, which alters its pharmacological properties. Furthermore, PYY 1–36 was found to bind to all Y receptor subtypes, whereas PYY 3–36 had a higher affinity for the Y2 subtype [13]. PYY plays multiple physiological roles in the gastrointestinal tract. PYY inhibits gastric secretion, delays the emptying of the stomach, stimulates water absorption, and regulates food intake and appetite [14]. Patients with inflammatory bowel disorders have altered levels of PYY. For instance, the density of PYY cells in the colon is lower in patients suffering from CD [15,16] and irritable bowel syndrome [17,18]. Similarly, the level of rectal PYY is lower in patients with ulcerative colitis [19]. Guided by these findings, we hypothesized that PYY plays functional roles in the pathogenesis of colitis.

To examine the possible role of PYY in IBD, we initially detected the expression of PYY and PYY receptors in the colon mucosae of mice with CD-like colitis induced by trinitrobenzene sulfonic acid (TNBS). Because PYY 3–36 is the main circulatory form of PYY [13], we investigated its regulatory role to influence LPS-induced macrophage cytokine production in vitro. We evaluated the in vivo effect of PYY 3–36 on colonic inflammation using the change in proinflammatory cytokine levels and colonic mucosal histopathological examination. Furthermore, we examined the proportion of Th1 and Th2 cells in the spleen by flow cytometry to explore the possible mechanism.

## Materials and methods

### TNBS-induced colitis mouse model and PYY3–36 treatment

Animal experiments were conducted in agreement with the European Convention for the Protection of Vertebrate Animals used for Experimental and Other Scientific Purposes. Ten- to twelve-week-old female mouse littermates on the Balb/c background were provided in specific pathogen-free conditions by the Center of Experimental Animals of Bethune Medical College of Jilin University (Changchun, China). The mice were housed in an environment with a 12-hour light–dark cycle with constant humidity and temperature and allowed free access to food and water (except during some experiments; see below). To produce the colitis disease model, food was removed for 24 h to decrease the effect of feces on performing rectal perfusion; colitis was induced by administering 2 mg of TNBS (Sigma, St. Louis, MO, USA) in 50% ethanol per 20 g of body weight by rectal perfusion. The evolution of the disease process was scored by survival rate, weight variation, myeloperoxidase (MPO) activity, damage to colon morphology, and cytokine measurement in colonic mucosa, macrophages, and serum.

As for the PYY3-36 treatment, we performed the protocol described in [20]. Mice (n ≥ 8 in each group) with CD-like colitis received 100 μl of 0.1 nM or 10 nM PYY 3–36 (Phoenix Biotech, Cat. No. 059–06, USA) per 20 g of each mouse by intraperitoneal (i.p.) injection. Mice were sacrificed on day 10 at the first-round experiment for evaluating the change of body weight and survival rate. At the second round experiment, mice was sacrificed on days 3 as shown in the Table 1. Serum and colon tissues were collected and stored until used for analysis.

**Table 1.** Experiment design for PYY3-36 treatment

Day	−1	0	1	2	3
Control	Water+ Starvation	Water	Water	Water	Sacrificed
Vehicle		Ethanol	Water	Water	
TNBS		Ethanol + 2 mg TNBS	Water	Water	
TNBS+ PYY3-36(0.1 nM)		Ethanol + 2 mg TNBS	PYY3-36	PYY3-36	
TNBS+ PYY3-36(10 nM)		Ethanol + 2 mg TNBS	PYY3-36	PYY3-36	

### Macrophages

To investigate the anti-inflammatory role of PYY 3–36, peritoneal macrophages were isolated from normal mice that had been intraperitoneally injected with 4 mL of 3% thioglycolate broth daily for 4 days. The cells were suspended (1 × 10^6^ per mL) in RPMI1640 medium containing 10% fetal bovine serum and 1% penicillin and streptomycin (complete medium). The nonadherent cells were removed after 2 h incubation at 37°C and 5% carbon dioxide (CO_2_). The remaining adherent cells were cultured overnight until they were treated with 1 μg/ml LPS in the presence of different working concentrations of PYY 3–36: 0nM, 0.01 nM, 0.1 nM, 1 nM, 10 nM, and 100 nM. After 6 h, the supernatant and cells were collected and stored at −80°C until used for analysis.

### Splenocytes for flow cytometry

Splenocytes were obtained from mice with CD-like colitis treated with or without 10 nM PYY 3–36 and purified as described [9]. The cells were incubated in complete medium at a density of 10^6^ cells per mL in a 37°C, 5% CO_2_ incubator. After incubating for 2 h, nonadherent cells were removed and the splenocytes were harvested and washed three times with phosphate-buffered saline (PBS). For intracellular staining, cells were stimulated with a mixture of 20 ng/ml phorbol 12-myristate 13-acetate, 1 μg/ml ionomycin, and 1 μg/ml brefeldin A for 5 h. Cells were washed and kept in PBS for flow cytometric analysis.

### Reverse transcriptase quantitative polymerase chain reaction (RT-qPCR)

Total mRNA was isolated from macrophages and colon mucosa, quantified, and reverse transcribed into complementary DNA. PCR was performed using a PCR kit (Takara, USA). Glyceraldehyde 3-phosphate dehydrogenase (GAPDH) was used as an endogenous reference gene. The primers designed for mice are shown in Table 2. The brightness of the PCR products was determined with Quality One software. Gene expression was normalized to GAPDH.

**Table 2.** Forward (F) and reverse (R) Primers

GENE	SEQUENCE	SIZE (bp)	Tm (°C)
PYY-F	5’-AACCAGAGGCTCCCGGCGA −3'	101	56
PYY-R	5'-TCCATACCGCTGCCGGGTGA-3'		
Y1-F	5'-GCAGGCTAGCCCAGTCGCAT-3'	166	56
Y1-R	5'-CCAAGCGCGCCTCATTCCGT-3'		
Y2-F	5'-CGCAAGAGTCAATACAGCCAAGTGA-3'	165	56
Y2-R	5'-TGAGCTCCGGCTCCGGATCAG-3'		
Y4-F	5'-GAGGGCGCTGCTTGGCTGAG-3'	152	56
Y4-R	5'-ATGCAGCCTGGCGAGGTACC-3'		
Y5-F	5'-CCAGGGCATCCCGAGGACTCTAG-3'	105	56
Y5-R	5'-GGAGGCCGTGTTCTGACTGGC-3'		
Y6-F	5'-AGTGACTCCAACTCCAGGGAATAGC-3'	220	56
Y6-R	5'-GTCAGCATGCTCTTGAGATCCTGTT-3'		
GAPDH-F	5'-ACCACAGTCCATGCCATCAC-3'	452	56
GAPDH-R	5'-TCCACCACCCTGTTGCTGTA-3'		
TNF-α-F	5'-CTGTGAAGGGAATGGGTGTT-3'	384	58
TNF-α-R	5'-CAGGGAAGAATCTGGAAAGGTC-3'		
IL-6-F	5'- CCAGAAACCGCTATGAAGTTCC-3'	486	55.5
IL-6-R	5'-GTTGGGAGTGGTATCCTCTGTGA-3'		

### Enzyme-linked Immunosorbent Assays (ELISAs) for MPO, PYY, TNF-α, and IL-6

The mouse MPO ELISA kit was purchased from Abcam (Cat. No. ab275109), PYY ELISA kit from Aviva Systems Biology (Cat. No. OKCD05062, USA), mouse TNF-α and IL-6 ELISA kits from Biolegend (Cat. No. 431,301 and 430,901, USA). The protocols were performed as the manufacturers described. In brief, 96-well plates were coated with the corresponding capture antibody. After incubation with blocking buffer (PBS + 1% bovine serum albumin), a 100-µl aliquot of either standards, serum, or colonic protein extracts (5 mg tissue per mL PBS supplemented with protease inhibitors) was added into each well individually and the plates were incubated for 2 h at room temperature. The detector antibody was added to the wells, the plate was incubated, and unbound detector antibody was removed by washing. The avidin-peroxidase conjugate was added, the plate was incubated, and unbound conjugate was removed by washing. The enzymatic reaction catalyzed by horseradish peroxidase (HRP) was initiated by adding the substrate, 3,3’,5,5’ tetramethylbenzidine dihydrochloride (TMB). The absorbance was measured by using a microplate reader at a wavelength 450 nm after the reaction was stopped with 2 M H_2_SO_4_.

### Protocol for flow cytometry

CD4^+^ T cells found among the isolated splenocytes were labeled with fluorescein-isothiocyanate (FITC)-conjugated anti-mouse CD4^+^ antibodies (Biolegend) and stained with phycoerythrin (PE)-labeled anti-mouse interferon-γ (IFN-γ) or IL-4 monoclonal antibodies (Biolegend) for 30 min at a cold temperature. Flow cytometric analysis was performed with FlowJo software (FlowJo LLC, Ashland, Oregon).

### Statistical analysis

All experimental data were statistically analyzed and compared using a t-test with Welch´s correction in GraphPad Prism 8. Student’s t-test was used elsewhere. P values ≤ 0.05 were considered statistically significant.

## Results

In this study, we investigated the potential role of PYY3-36 in CD-like colitis induced by TNBS. The expression of PYY and PYY receptors in the colon mucosae of mice were detected. The anti-proinflammatory role of PYY-36 was examined with LPS-induced macrophage in vitro. The effect of PYY 3–36 on colonic inflammation was determined by using the change in proinflammatory cytokine levels and colonic mucosal histopathological examination. The possible mechanism was explored by the examination of the proportion of Th1 and Th2 cells in the spleen.

### The expression of PYY and Y Receptors in mice with TNBS-induced colitis

**Figure 1.**
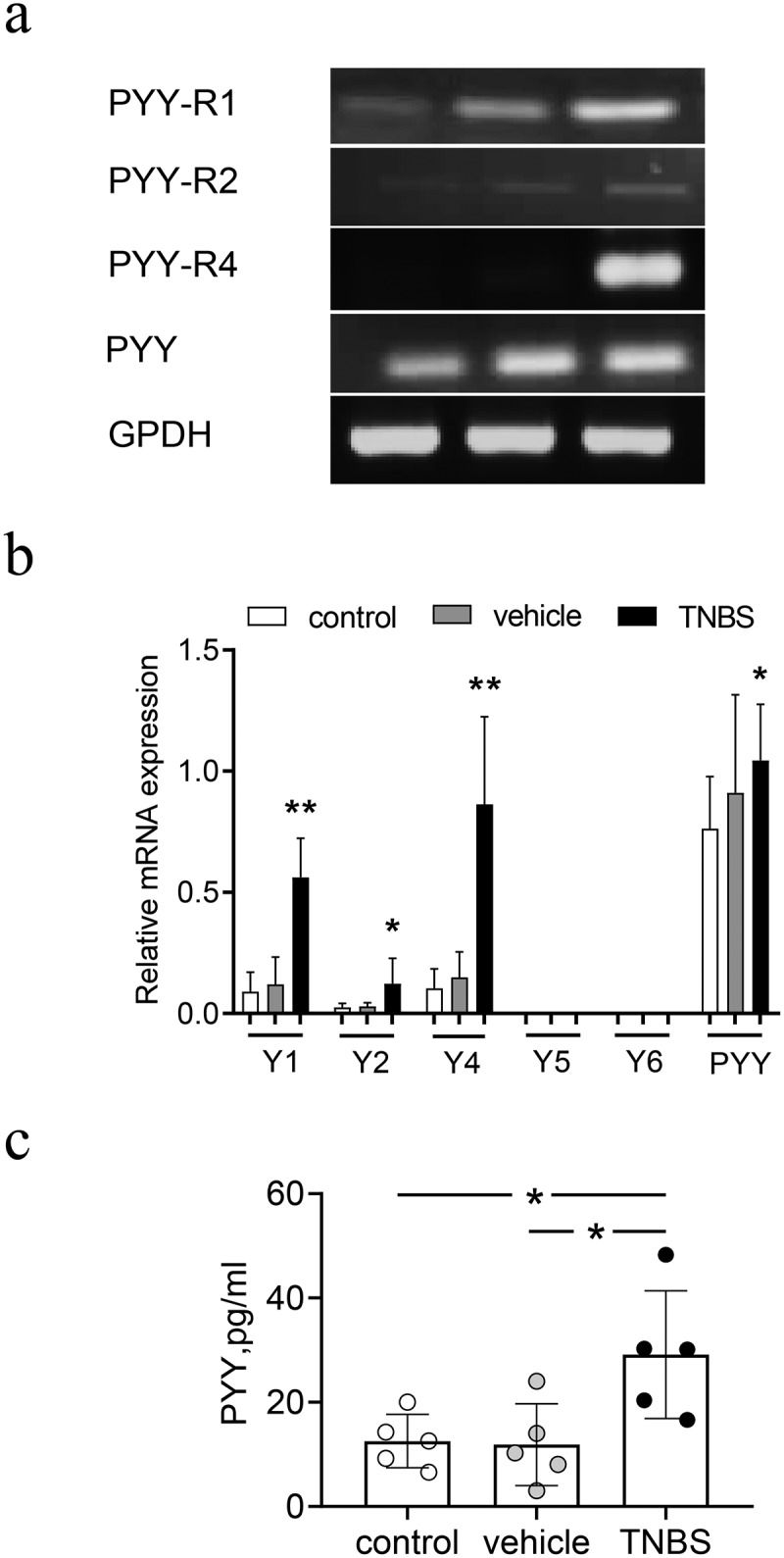
**Expression of PYY and PYY receptors in mice with TNBS-induced colitis**. Balb/c mice (n = 6 for each group) were used to establish a model of colitis induced by intracolonic administration of TNBS (2 mg per 20 g of body weight) mixed with 50% ethanol. All mice were sacrificed on day 3 after administration of TNBS. The colon mucosa was collected to extract its RNA. (a) The expression for PYY and its receptors Y1, Y2, Y4, Y5, and Y6 was measured using quantitative PCR. (b) The brightness of the PCR products was scanned using Quality One software (Data are presented as Mean ±SD). (c) The expression of PYY proteins in the colon mucosa was determined by ELISA. Each dot represents one mouse. n ≥ 5 mice per group; *, p < 0.05; **, P < 0.01; ***, P < 0.001 versus control mice.

To determine whether PYY was involved in gastrointestinal injury, we initially established a CD-like mouse model in which colitis was induced by TNBS. We isolated mRNA from colon mucosa to measure the expression of PYY and its five different PYY receptors Y1, Y2, Y4, Y5, Y6 with quantitative PCR. As shown in Figure 1(a, b), no expression of Y5 and Y6 in mucosa colon was found. In contrast, the expression of PYY and receptors Y1, Y2, and Y4 in mice with TNBS-induced colitis was significantly higher than that in mice treated with vehicle (Figures 1(a, b)). In addition, PYY protein level in mice with TNBS-induced colitis was significantly higher than the other two groups (Figure 1(c)). This suggests that not only PYY, but also Y1, Y2, and Y4, participate in experimental colitis.

### High concentrations of PYY 3–36 inhibits the production of inflammatory mediators in activated macrophages in vitro

Based on the findings above, we examined whether PYY 3–36 has an anti-inflammatory role by determining the effect of different concentrations of PYY 3–36 on the production of proinflammatory cytokines TNF-α and IL-6 from peritoneal macrophages activated by lipopolysaccharide (LPS). All concentrations of PYY 3–36 from 0.1 nM to 100 nM significantly inhibited TNF-α production (Figure S1a), whereas only 10 nM and 100 nM of PYY 3–36 significantly inhibited IL-6 production (Figure S1b).

### PYY 3–36 ameliorates inflammation in mice with TNBS-Induced colitis

**Figure 2.**
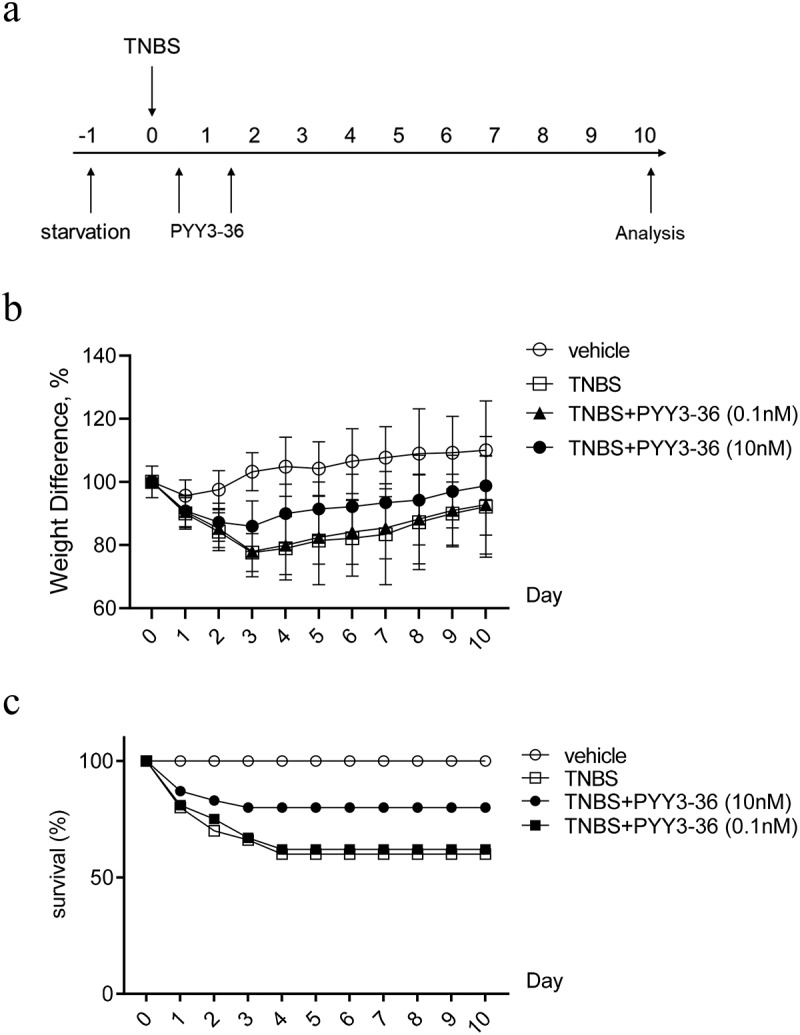
**PYY 3–36 treatment improves the weight loss and death rate of mice with TNBS-induced colitis**. Mice treated with 50% ethanol in PBS were used as the vehicle control. Colitis was induced by TNBS (2 mg per 20 g of body weight) at day 0. After 12 h, mice received intraperitoneal injections of 0.1 nM or 10 nM PYY 3–36. (a) All experimental Balb/c mice were weighed every day and sacrificed on day 10. (b) Weight change normalized to weight data on day 0 and (c) survival rate was calculated to assess the disease progression. Three individual experiments were performed n ≥ 5 mice in each group.

**Figure 3.**
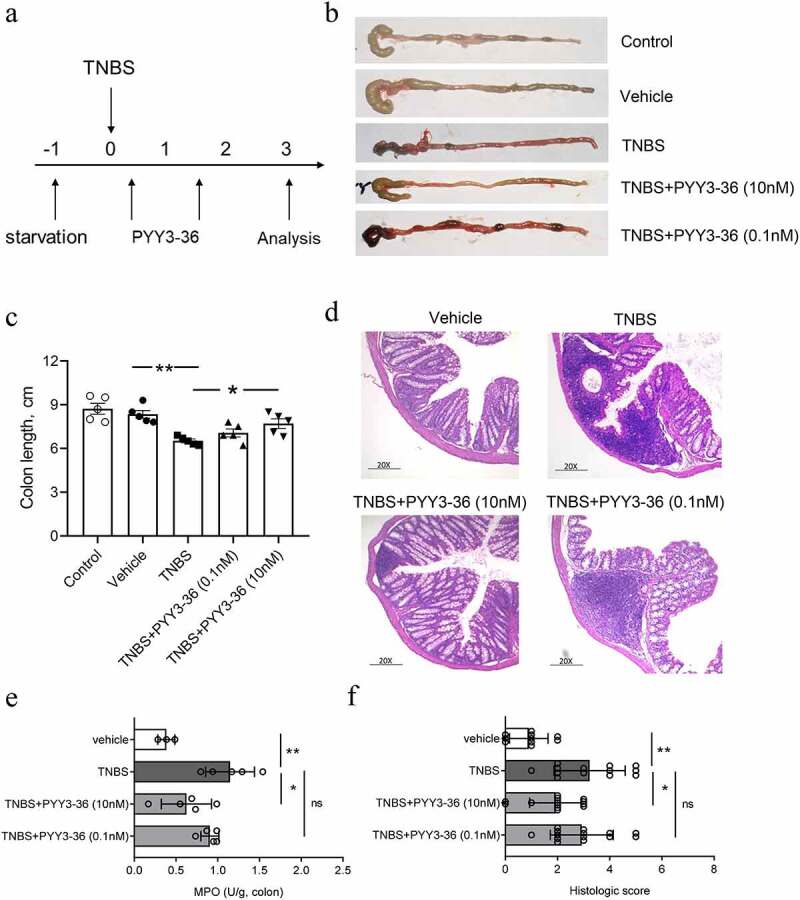
**PYY 3–36 treatment abrogates the development of TNBS-induced colitis**. Mice treated with 50% ethanol in PBS were used as the vehicle control. Colitis was induced by TNBS (2 mg per 20 g of body weight) at day 0. After 12 h, mice received intraperitoneal injections of 0.1 nM or 10 nM PYY 3–36. (a) All experimental Balb/c mice were weighed every day and sacrificed on day 3. Clinical evolution and severity were assessed by (b) macroscopic observation, (c) colon length, (f) histologic score of (d) hematoxylin and eosin-stained sections, and (e) myeloperoxidase (MPO) level in colon tissues at day 3 after TNBS administration (Original magnification 20×) *, p < 0.05; **, p < 0.01 versus the mice injected with TNBS.

Because 10 nM PYY 3–36 effectively suppressed the production of inflammatory mediators, we next examined whether that concentration has therapeutic effects on TNBS-induced colitis in mice. We first monitored the weight and survival data of all experimental mice until day 10 after administration of TNBS. The mice lose weight until day 3 and then recover until day 10, when they were sacrificed (Figure 2(b)). Mice that received intrarectal administration of TNBS experienced a 64% mortality. In contrast, the survival rate increased to around 80% for TNBS-instilled mice treated with 10 nM PYY 3–36, whereas 0.1 nM PYY 3–36 had no effect (Figure 2(c)). Similarly, macroscopic observation revealed that the colons of mice treated with TNBS had striking inflammation compared to mice treated with vehicle only, which had no inflammation (Figure 3(b)). In contrast, the colons from TNBS+PYY 3–36 treated mice showed signs of strongly diminished macroscopic inflammation (Figure 3(b, a)) striking recovery of colon length (Figure 3(c)). Histological observation of colons from TNBS-instilled mice showed epithelial cell loss, patchy ulceration, red blood cell increases, and infiltration of inflammatory cells, such as neutrophils, lymphocytes, and macrophages (Figure 3(d–f)). Increased myeloperoxidase (MPO) activity in the colon was correlative with neutrophil infiltration (Figure 3(e)). In summary, when mice instilled with TNBS were treated with PYY 3–36, the histological and macroscopic signs were strikingly improved and neutrophil infiltration and inflammatory activity significantly decreased.

### PYY 3–36 reduced the systemic level of TNF-α and IL-6 in mice with TNBS-Induced colitis

**Figure 4.**
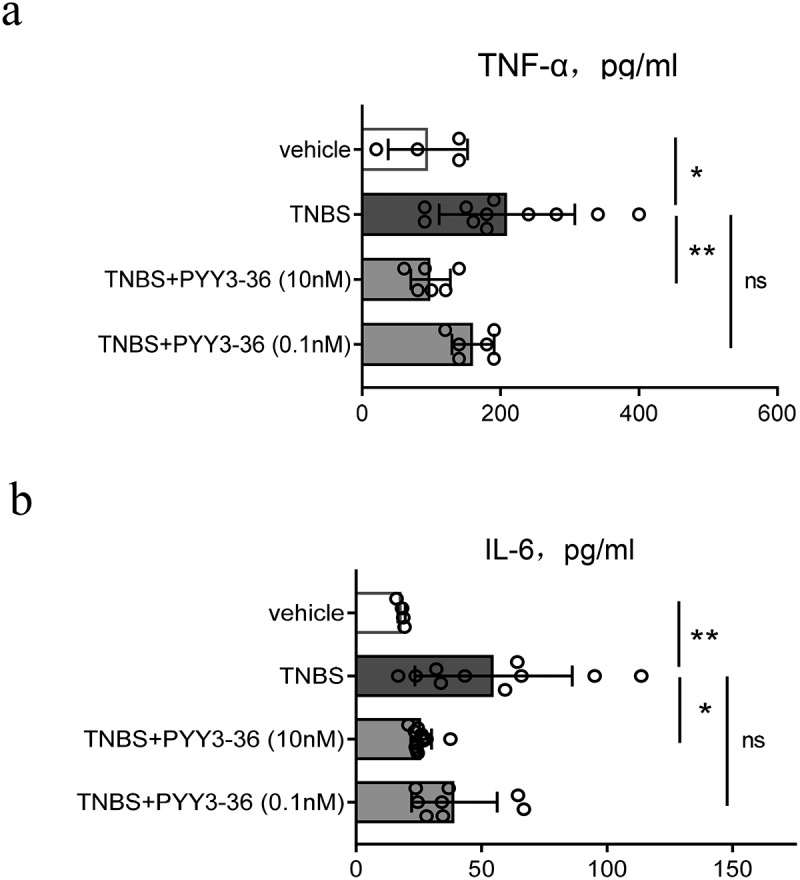
**PYY 3–36 treatment reduces the level of serum TNF-α and IL-6 in mice with TNBS-induced colitis**. Colitis was caused by TNBS via intracolonic administration. Mice treated with 50% ethanol in PBS as the vehicle control. Mice were injected i.p. with 0.1 nM or 10 nM PYY 3–36 12 h after TNBS instillation. Serum was obtained from the collected blood after sacrificing control and experimental mice at peak of disease progression on day 3. TNF-α and IL-6 were determined by ELISA. *, p < 0.05 **, P < 0.01 versus the mice injected with TNBS.

To investigate the potential preventive influence of PYY 3–36 on colitis caused by TNBS in mice, we collected the serum at the peak of disease progression on day 3 and evaluated whether PYY 3–36 affected production of inflammatory cytokines TNF-α and IL-6 correlating with the mechanism of colitis induced by TNBS. PYY 3–36 significantly reduced the level of systemic proinflammatory cytokines TNF-α and IL-6 (Figure 4(a, b)). This suggests a strong potential for PYY 3–36 to modulate the production of cytokines in colonic inflammation.

### PYY 3–36 inhibited the production of TNF-α and IL-6 in the colons of mice with colitis

**Figure 5.**
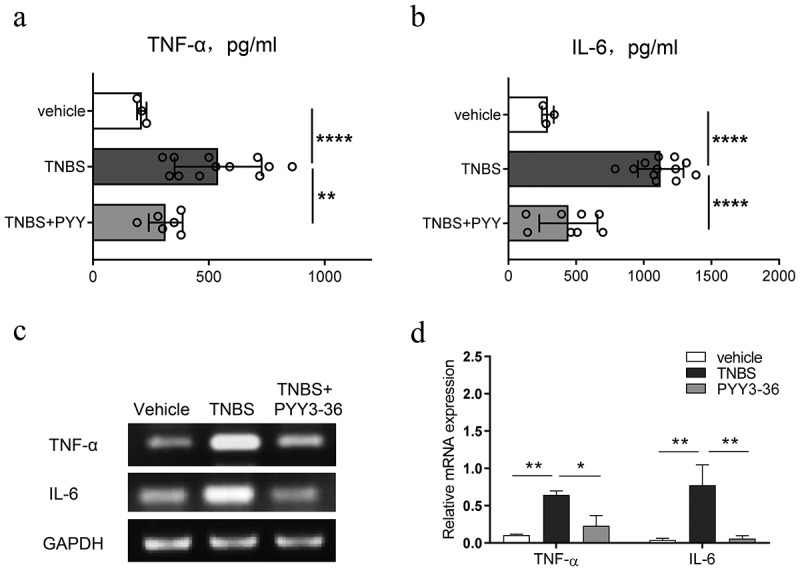
**PYY 3–36 inhibited the production of TNF-α and IL-6 in the colons of mice with colitis**. Mice treated with 50% ethanol in PBS were used as the vehicle control. Colitis was induced by TNBS (2 mg per 20 g of body weight) at day 0. After 12 and 36 h, respectively, mice received i.p. injections of 10 nM PYY 3–36. After 3 d, mice were sacrificed and the colon tissues were collected. The tissues were used for the measurement and gene expression of TNF-α and IL-6 by ELISA (a and b) or PCR (c and d). *, p < 0.05 **, P < 0.01 ***, P < 0.001 ****, P < 0.0001 versus TNBS-injected mice.

To further strengthen the demonstrated anti-inflammatory action of PYY 3–36, we sampled colon tissue from normal and TNBS-injected mice with or without PYY 3–36 (10 nM) on day 3 after TNBS administration. We determined the level of TNF-α and IL-6 in colon tissue. PYY 3–36 inhibited the production of TNF-α and IL-6 (Figure 5(a–d)), suggesting that PYY 3–36 exerts protective role through inhibition of the inflammatory mediators TNF-α and IL-6.

### PYY 3–36 modulates the differentiation of splenic Th1/Th2 Cells in TNBS-induced colitis

**Figure 6.**
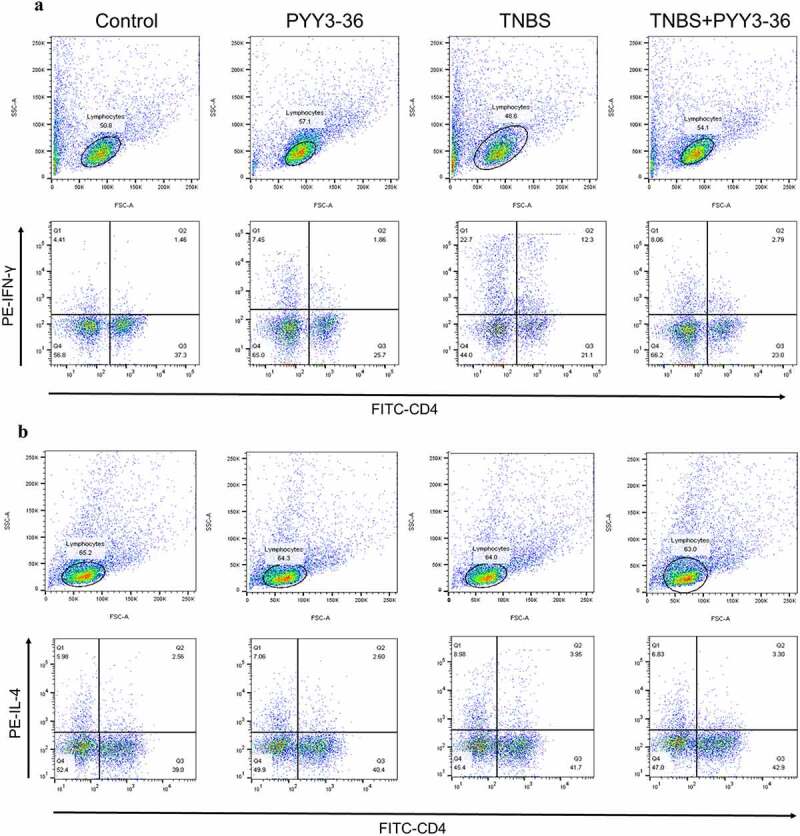
**Modulations of IFN-γ- or IL-4-producing CD4^+^ T cells induced by PYY 3–36**. Splenocytes were isolated from normal mice with or without pretreatment with 10 nM PYY 3–36 for 2 h and from TNBS-injected mice with or without pretreatment with 10 nM PYY 3–36. The splenocytes were *ex vivo* stimulated with PMA (20 ng/ml), ionomycin (1 μg/ml), and brefeldin A (1 μg/ml). Flow cytometry was performed to quantify CD4^+^ T cells producing IFN-γ (a) and IL-4 (b).

CD is a Th1 cell-mediated disease characterized by increased levels of proinflammatory cytokines IFN-γ, TNF-α, and IL-6, increased levels of Th1 cytokines IL-2 and IL-12, and minor changes in Th2 cytokines IL-4 and IL-10 [21]. To address the mechanism of the protective role of PYY 3–36, we examined its effect on the differentiation of Th1/Th2 cells in splenocytes. Spleen cells were isolated from experimental mice, and then were stained with CD4^+^, IFN-γ, and IL-4 fluorescent antibodies for flow cytometry. The percentage of IFN-γ- producing CD4^+^ T cells was strikingly increased in the spleens of TNBS-treated mice (Figure 6a). However, this increase was reduced by the administration of PYY 3–36(10 nM) (Figure 6b). Interestingly, we observed a minor increase in the percentage of IL-4-producing CD4^+^ T cells in the colitis mouse model (Figure 6(a)), which was also reduced slightly by PYY 3–36 (Figure 6b). The ratios of Th1/Th2 CD4^+^ cells in TNBS-treated mice with or without treatment of PYY 3–36 are markedly different. More precisely, PYY 3–36 not only lowered the percentage of splenic Th1 and Th2 cells but also reduced the proportion of Th1/Th2 cells (Figure 6(a, b)). These results indicate that PYY might attenuate CD by modulating the differentiation of Th1/Th2 cells.

## Discussion

Currently, the relatively ideal strategies for the treatment of IBD are to block its progression to bowel damage, complication, and disability. Usually, no ulcerations through endoscopic healing are the major therapeutic target in IBD, but it only reduces relapse rates, bowel damage, and the need for surgery [22]. In addition, although many therapeutic agents are used for treating IBD, they are either not that effective or have non-negligible side effects [2]. PYY levels are altered during the development of IBD and PYY 3–36 is the major form in circulation [13], indicating that it might play an important role in CD. Therefore, we established and utilized the TNBS-induced colitis CD-like mouse model to address whether PYY is involved in CD and determine its potential role CD. Consequently, we found PYY 3–36 had a protective role in mice with TNBS-induced colitis and this protection might be through inhibiting the production of proinflammatory cytokines TNF-α and IL-6 from activated macrophages and by modulation of the balance of Th1/Th2.

PYY binds to six transmembrane G protein-coupled receptors Y subtypes, namely Y1-Y6 (although Y3 has not been cloned) [23–25]. A recent study reported that PYY is involved in signaling in the lateral parabrachial nucleus to increase food intake though Y1 in mice [26]. The Y2 receptor is thought to be intrinsically involved in the regulation of energy balance by PYY [27]. Y4 mediates the inhibitory action of PYY on enterocytes [28]. No functional roles have been found for Y5 and Y6 in colonic epithelium through binding with PYY. Here, we found only Y1, Y2, and Y4 were expressed; their expression in mice with colitis was significantly higher than that of control mice. These results demonstrate that Y1, Y2, and Y4, as well as PYY, may play important roles in the experimental CD-like mouse model. Interestingly, Y1, Y2, and Y4 expression almost doubled in the TNBS-treated group compared to that in the control group, whereas PYY expression was only slightly higher. Which indicates the binding ability for PYY with its receptors is probably suppressed. Additionally, the other two peptides from same family, neuropeptide Y (NPY) and pancreatic polypeptide, bind to the same receptors as PYY and may also play some unknown functional roles in the experimental colitis mouse model. Therefore, more studies should be performed to provide more insight into the roles of Y1, Y2, and Y4 in colitis.

The TNBS-induced disease model has been used extensively to examine various aspects potentially related to CD. TNBS induces experimental colitis with remarkable clinical symptoms characterized by severe granulomatous and transmural inflammation identical to that observed in human CD [29]. Therefore, it is valuable to improve the severity of disease and provide relief in inflammation in this CD-like mouse model from a therapeutic viewpoint. In our finding, PYY 3–36 strongly mitigated the loss of body weight, colonic inflammation, and MPO activity and increased the survival rate in mice with TNBS-induced colitis.

Numerous studies have reported that proinflammatory cytokines and immune cells play pivotal roles in CD [21]. Colitis induced by TNBS is a disease mediated by the activation of Th1 cells and subsequent macrophage recruitment and activation [29]. TNF-α, IL-6, and some other cytokines are crucial in the establishment of chronic inflammation. Therefore, some studies have suggested blocking those inflammatory meditators as therapy targets thereby to restrain the development of gastrointestinal diseases [29–35]. Based on this evidence, a recent study reported that mycophenolate mofetil could be used to treat patients with IBD because it down-regulates the protein and mRNA levels of those cytokines listed above as being increased in TNBS-treated mice [9]. Two promising candidates, cortistatin and baicalein, exert similar immunomodulatory effects as mycophenolate mofetil in improving CD in the mouse model like [36,37]. Macrophages play pivotal roles in colonic inflammatory responses by releasing cytokines and chemokines, and by presenting antigens to T cells [38]. In intestinal inflammation, the activated macrophages contribute to the secretion of cytokines, such as TNF-α, IL-6, IL-23, and IL-1β and promote Th1 and/or Th17 cell immune responses [39]. To gain mechanistic insight, we utilized isolated peritoneal macrophages to explore the potential anti-inflammatory role of PYY 3–36. In our study, PYY 3–36 shows similarly striking inhibition on not only the level of systemic proinflammatory cytokines TNF-α and IL-6 but also the production of TNF-α and IL-6 from peritoneal macrophages activated by LPS or from colon tissues of CD-like mice treated with PYY 3–36. Furthermore, the expression of TNF-α and IL-6 in peritoneal macrophages from mice with colitis was down-regulated by a high concentration (10 nM) of PYY 3–36. These results indicate that PYY 3–36 might alleviate colonic inflammation though lowering the production of TNF-α and IL-6 by activated macrophages.

To date, the M1 and M2 macrophage ratio and Th cell-mediated immune responses (especially Th1/Th2 and Th17/Treg) play important roles in the regulation of gut microbiota in the pathogenesis of IBD. Gut microbiota is different in patients with IBD compared to that in healthy controls. Altered bacterial populations in the colon were found to be correlated with increased disease activity [40]. Therefore, in current studies the modulation of colonic bacterial populations was used as a reference to evaluate the functional role for agents in experimental colitis. A very recent study showed PYY deficiency in mice disturbed the gut microbiome composition in response to a high-fat diet [41], indicating PYY 3–36 and gut microbiota might have potential correlation in colitis. The M1 macrophage is proinflammatory, whereas the M2 macrophage is anti-inflammatory; they are both located throughout the gastrointestinal tract. The polarization between M1 and M2 macrophages is of great importance in the progression and prognosis of inflammation [42]. In CD, switching the imbalance of M1 and M2 has become the key strategy to alleviate colonic inflammation [43–45]. Here, we found PYY 3–36 reduced the secretion and expression of macrophage-derived TNF-α and IL-6, the M1 macrophage cytokines, suggesting PYY 3–36 might shift the M1/M2 ratio. Further studies for these speculations are needed.

The immunological pathogenesis of IBD is also pivotal in the disease progression, especially T helper cell-mediated immune responses. However, the detailed mechanism remains unknown. It has been proposed to classify CD4^+^ T cells into Th1, Th2, Th9, Th17, Th22, Tfh, Treg, and Tr1 cells. They all have been reported to play a crucial role in the development of IBD [46]. Th17/Treg and Th1/Th2 cell imbalances were found to be crucial for the development of IBD, so correction of this imbalance was considered a target to prevent and treat IBD. For instance, the use of arbutin or IL-12/IL-23p40 peptide-based vaccines could ameliorate the TNBS- or dextran sulfate sodium (DSS)-induced ongoing chronic inflammation and fibrosis by rebalancing Th1/Th17/Treg responses [47,48]. Studies revealed that phytochemicals such as arctigenin, cambogin, and icari improve the inflammation of inflammatory bowel diseases by regulating Th17/Treg balance [49]. A recent report showed the imbalance of Th1/Th2 was corrected by *Inonotus obliquus* polysaccharide in mice with DSS-induced colitis [50]. The balance of Th1/Th2 responses, as the classical dichotomy, is widely believed to be crucial for the development of CD, and its importance was demonstrated by many studies. Medications such as cortistatin, baicalein, and mycophenolate mofetil used for treating IBD in the clinic were found to attenuate the development of CD in a mouse model by modulation of Th1/Th2 differentiation [9,36,37]. Thus, in our study, we utilized this mechanism to address the potential role of PYY 3–36 in the CD model. Interestingly, we found PYY 3–36 also has a modulatory role in the differentiation of Th1/Th2 cells.

This study preliminarily unearths that the therapeutic effects of PYY 3–36 on experimental CD may occur via inhibiting the release of TNF-α and IL-6 from macrophages and correcting the imbalance of Th1/Th2. However, more studies need to be done to verify this ameliorative role of PYY 3–36 using mouse models of colitis induced by other chemical agents like DSS and oxazolone [51]. Furthermore, we only investigated the anti-inflammatory role for PYY 3–36 at one time (day 3); it would be interesting to study other time points to track PYY 3–36 functions over time. Even though there are limitations mentioned above, our study provides the foundation to further study the protective role of PYY 3–36 in colitis.

## Conclusions

In summary, our study demonstrated the attenuation of TNBS-induced colonic inflammation, reflected by the improvement of weight loss, the reduction of immune cell infiltration, and proinflammatory cytokines expression, when mice are treated with a high concentration of PYY 3–36. A potential mechanism is the inhibition of macrophages producing the inflammatory mediators TNF-α and IL-6 and shifting the Th1/Th2 balance by PYY 3–36.

## Data Availability

The data supporting the findings in our study are available from the both submitting and corresponding authors upon reasonable request.
